# Paediatric and neonatal sepsis and inflammation

**DOI:** 10.1038/s41390-021-01918-4

**Published:** 2022-01-19

**Authors:** E. J. Molloy, C. F. Bearer

**Affiliations:** 1 Paediatrics, Trinity College Dublin, Trinity Research in Childhood Centre (TRiCC), Dublin, Ireland; 2 Trinity Translational Medicine Institute (TTMI), Dublin, Ireland; 3 Neonatology, Coombe Women’s and Infants University Hospital, Dublin, Ireland; 4 Neonatology, CHI at Crumlin, Dublin, Ireland; 5 Children’s Hospital Ireland (CHI) at Tallaght, Dublin, Ireland; 6 UH Rainbow Babies & Children’s Hospital, Cleveland, OH USA; 7 Case Western Reserve University School of Medicine, Cleveland, OH USA

## Abstract

Sepsis has a huge impact on global mortality and has been declared as a priority by the World Health organisation the WHO.^1^ Children have a high incidence of sepsis especially in the neonatal with an estimated 3 million babies affected worldwide and mortality ranges from 11 to 19%.^2^ In addition, long-term neurodevelopmental outcomes are affected but this is largely unquantified. However, challenges remain in the early recognition, diagnosis and standardised management of sepsis. This series on Sepsis and inflammation in children reviews the conundrums of diagnostic criteria, biomarkers, management and future strategies to improve outcomes.

## Definitions, diagnosis

The Sepsis-3 consensus defined adult sepsis as a “life-threatening organ dysfunction caused by a dysregulated host response to infection”.^3^ The Paediatric sepsis definition is under development by the International Pediatric Sepsis Definition Taskforce and has found strong associations of organ dysfunction markers linked with clinical outcomes suitable for inclusion in the validation phase of the definition.^4^ However neonatal sepsis definitions are not aligned with those in adults and children as many clinicians still rely on microbiological results rather than organ dysfunction.^5,6^ In addition to the lack of an internationally accepted consensus definition of neonatal sepsis, there are no long-term outcomes or core outcome datasets to standardise clinical trials of sepsis and allow comparison between trials.^7^

The Surviving Sepsis Campaign guidelines highlighted the need for research in clinical trials on paediatric sepsis recognition and QI screening tool algorithms to recognise clinical deterioration as essential.^8^ Rapid development in sepsis recognition tools have occurred in recent years and involve the electronic health record. Eisenberg et al. describe the different sepsis screening tools that have evolved and discuss the risk of alarm fatigue if too many false positives are detected.^9^

Celik et al. discuss the issues related to the diagnosis of neonatal sepsis as culture methods alone are not adequate and traditional laboratory biomarkers such as CRP and PCT have limitations as serial measurements are required.^10^ Molecular diagnostic tools such as PCR and sequencing, and mass spectrometry offer promise for more rapid and sensitive detection of disease but are not yet in routine clinical practice. Machine learning, AI and multi-omics may allow future personalised care and rapid early accurate diagnosis of sepsis.

## Biomarkers

Sullivan et al. described the use of vital signs in the diagnosis and as biomarkers of paediatric sepsis.^11^ mechanisms of vital sign changes in neonatal sepsis, including the cholinergic anti-inflammatory pathway mediated by the vagus nerve and the systemic inflammatory responses signalling the autonomic nervous systems. Sundararajan et al. suggested that future research should concentrate on developing machine learning models that utilise bedside vital sign datasets with standardised analytical methods.^12^

Buonsenso et al. described newer tools to discriminate between viral and bacterial sepsis in febrile children.^13^ Transcript host RNA signatures may allow to better characterise the spectrum of viral, bacterial and inflammatory illnesses in febrile children and can be used with traditional diagnostic methods to determine if and when to start antibiotic therapy.

Wong describes paediatric sepsis biomarkers including the discovery and development of diagnostic biomarkers for sepsis.^14^ Biomarkers allow stratification of patient subgroups for individualised therapy and also act as markers of response to therapy. The Pediatric Sepsis Biomarker Risk Model (PERSEVERE) incorporates a panel of serum protein biomarkers, measured within 24 h of a sepsis diagnosis, to estimate baseline risk of mortality among critically ill children with septic shock. The PERSEVERE biomarkers appeared to have utility for estimating the risk of poor functional and quality of life outcomes among children who survived the acute stage of sepsis and were discharged from the hospital. This was also corroborated in an experimental mouse model of sepsis and has confirmed the reliability of these models employing artificial intelligence and transcriptomic data.^15^ Three endotypes of sepsis termed inflammopathic, adaptive, and coagulopathic, have been defined relating to their respective endotype-defining gene expression signatures. The coagulopathic endotype was associated with the highest mortality rate among the three endotypes. Paediatric septic shock endotypes A and B are defined by 100 genes corresponding to the adaptive immune system and glucocorticoid receptor signalling. Endotype A is independently associated with higher mortality and organ failure burden.^14^ It is suggested that combining biomarkers with endotypes may improve these predictive models.

Issues with common biomarkers are described by Tiozzo et al. especially in the understanding of false-positive test results is equally relevant in optimising use.^16^ This group explored common patient characteristics and non-infectious conditions which change inflammatory biomarkers commonly used in the diagnosis of sepsis. They emphasised the need to incorporate patient outcomes and examined ways of improving biomarker performance in sepsis.

## Mechanisms and aetiology in sepsis

Chorioamnionitis in neonatal sepsis is well-delineated in the review by Jain et al. demonstrating the multiorgan effects on the fetus. Microbes are not commonly detected although a variety of organisms have been described and antibiotic therapy has not prevented morbidity and mortality. Persistent immune dysfunction has been described and linked with adverse outcomes with increased risk of sepsis and neurodevelopmental outcomes associated with altered immune programming in fetal life.^17,18^

Immune cell phenotypes are linked with epigenetics, including DNA methylation, histone tail modifications and microRNA expression and therefore they influence immune outcomes. Perinatal exposure alters epigenetic profiles of immune cells and can be targeted in neonatal sepsis.^19^ Perinatal factors such as maternal illness, obesity, sepsis and socioeconomic factors affect the epigenetic profiles. In sepsis pre-exposure with chorioamnionitis and postnatal sepsis alter immune cell responses. Herz et al. examine the role of peripheral immune cells in brain injury in preterm and term infants and possible immunomodulation.^20^

O’Reilly et al. highlight the important role of platelets in the immune response to sepsis in neonates and children.^21^ Platelets are mediators of haemostasis and thrombosis, but also interact with adaptive and innate immune cells. Although thrombocytopenia is well-recognised in paediatric sepsis more detailed research is warranted to understand the mechanisms of interactions with other immune cells such as neutrophils and monocytes.

Mithal et al. examined the need for further knowledge on the role of environmental and infectious exposures and age, co-morbidities prior exposures, and genetics on the pathophysiology of paediatric sepsis.^22^ Accurate immune phenotyping during sepsis and endotyping sepsis subtypes may be particularly important in children. Precision medicine techniques to individualise care and not use a one size fits all approach is essential in the management of paediatric sepsis.

Ryan et al. give an overview of the effect of SARS-Cov 2 on neonates with the emerging multisystem inflammatory syndrome in children (MIS-C). The COVID-19 pandemic had a significant effect on neonatal health, their quality of care and indirect influences on their clinical course.^23^ There is a need to ensure ongoing research in newborns and children regarding the effects of SAR-CoV-2.

## Treatments and future plans in paediatric and neonatal sepsis

Burns et al. reviewed the use and rationale for antibiotic choices in sepsis.^24^ Critically ill children can be harmed by inadequate or inappropriate antibiotic use and early recognition and management of sepsis are vital for improved morbidity and mortality. However, overuse of antibiotics affects the microbiome, contribute to organ dysfunction and may have idiosyncratic toxicities. The importance of antibiotic stewardship programmes and surveillance are key to appropriate antibiotic use in paediatric sepsis. Individualised dosing and duration of therapies guided by biomarkers response can minimise antibiotic use without increasing harm. In a recent Cochrane study, there was insufficient evidence to support any antibiotic regimen being superior to another in late-onset neonatal sepsis. The authors suggest further RCTs of different antibiotic regimens in late-onset neonatal sepsis are required.^25^ Flannery et al. the emergence of multidrug-resistant microorganisms requiring Surveillance and prevention is a paediatric research priority. Extended-spectrum β–lactamase (ESBL)-producing Enterobacterales and carbapenem-resistant Enterobacterales (CRE) are increasing and their impact on morbidity and novel antibiotic therapies are required.^26^

Fungal infections are relatively common in preterm infants and critically ill children. Weimar et al. highlight the increased risk in extremely low birth weight infants related to their decreased skin integrity, altered immune function and subsequent use of antibiotics.^27^ *Candida* spp. are the most common causative organisms but there are increasing reports of different species. Sepsis care bundles, minimising duration of central catheters, hand hygiene and antibiotic stewardship and prophylactic use of fluconazole in high-risk infants may prevent invasive fungal infections. Advanced time-domain bioinformatic datasets extracted from bedside telemetry data is to enrich the predictive monitoring system and assist the bedside clinician in successfully identifying infants at risk for sepsis and preventing morbidity and mortality.

Swigart et al. outline a multidisciplinary consult service to manage complex multiorgan dysfunction in children with sepsis and associated immune dysfunction including critical care, immunology, rheumatology, infectious disease, haematology, oncology, gastroenterology, and hepatology.^28^ This critical inflammation group have developed standardised consult plans for common consult types including SAI, hyperinflammation syndromes such as MAS and severe COVID-related multisystem inflammatory syndrome in children (MIS-C) with individualised care of these complex disorders. Kharrat et al. give an overview of cardiovascular dysfunction one neonatal sepsis. They propose the use of echocardiography guided management^29^.

Vitamins in high-dose have been suggested for the treatment of sepsis but studies to date have not been definitive including RCTs in high-dose vitamin C with no advantage.^30^ Vitamin D is also protective in respiratory illness in bronchiolitis and COVID-19.^31^

In conclusion, Kurul et al. demonstrated the knowledge gaps in paediatric and neonatal sepsis research.^32^ Increased collaborative research since the COVID pandemic and the intensive research in sepsis has the potential to improve child and neonatal outcomes in sepsis in the future.
